# Privacy-Preserving Location-Based Service Scheme for Mobile Sensing Data †

**DOI:** 10.3390/s16121993

**Published:** 2016-11-25

**Authors:** Qingqing Xie, Liangmin Wang

**Affiliations:** 1 School of Computer Science and Technology, Anhui University, Hefei 230601, China; xieqn@ahu.edu.cn; 2 School of Computer Science and Communication Engineering, Jiangsu University, Zhenjiang 212013, China

**Keywords:** mobile sensing, mobile cloud, location-based service, privacy preservation

## Abstract

With the wide use of mobile sensing application, more and more location-embedded data are collected and stored in mobile clouds, such as iCloud, Samsung cloud, etc. Using these data, the cloud service provider (CSP) can provide location-based service (LBS) for users. However, the mobile cloud is untrustworthy. The privacy concerns force the sensitive locations to be stored on the mobile cloud in an encrypted form. However, this brings a great challenge to utilize these data to provide efficient LBS. To solve this problem, we propose a privacy-preserving LBS scheme for mobile sensing data, based on the RSA (for Rivest, Shamir and Adleman) algorithm and ciphertext policy attribute-based encryption (CP-ABE) scheme. The mobile cloud can perform location distance computing and comparison efficiently for authorized users, without location privacy leakage. In the end, theoretical security analysis and experimental evaluation demonstrate that our scheme is secure against the chosen plaintext attack (CPA) and efficient enough for practical applications in terms of user side computation overhead.

## 1. Introduction

Recently, mobile sensing devices have been widely used in data sensing [1,2], including location data [3,4]. For example, when a person takes photos by a smart phone, the equipped location sensor (GPS modules) can always acquire the locations where the photos are taken [5]. Additionally, the locations are embedded into the photos for remembrance. Then, these location-embedded data will be published in the mobile cloud automatically [1,2,6], such as iCloud, Samsung cloud, etc. These location-embedded data bring great convenience for cloud service providers (CSP) to provide location-based services (LBS) for users [3,7].

**Figure 1:**
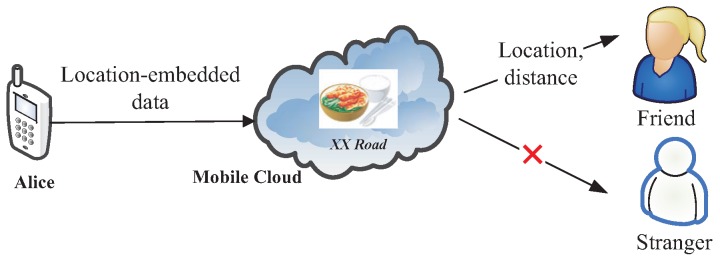
An example in which Alice shares location-embedded data with her friends.

However, the mobile cloud is untrustworthy. Additionally, the location itself contains much personal information [8,9,10]. CSP is curious to infer and analyze location data to harvest additional information to gain illegal profits. Thus, the publisher (i.e., data owner) requires a solution that can protect location privacy from unauthorized users and CSP. As shown in Figure 1, Alice takes some food photos by iPhone, and the photos are embedded with location information. She stores them in iCloud and shares them with her friends. Since the location where the photos are taken is her home, she hopes that the embedded location is visible only to her friends, while invisible to strangers.

A naive solution is to store the location data on the mobile cloud in an encrypted format. To achieve secure sharing on encrypted data, some researchers have studied the attribute-based encryption (ABE) scheme [4,11,12,13,14]. In this scheme, the publisher encrypts the confidential location information using a symmetric encryption scheme (AES or DES) and defines the access policies, then uploads the encrypted location and access polices into the cloud for storage. Only authorized queriers (whose attributes satisfy the access policies) can read the location data. During the whole procedure, the cloud undertakes only storage overhead. However, in LBS, the queriers also require the CSP to provide the services of location distance compute and compare. By applying ABE directly, the authorized queriers cannot process the location until downloading and decrypting the encrypted location data. It takes the queriers too much local storage and computation cost, which is unacceptable considering the weak power of smart phones.

To support functional processing over encrypted data, homomorphic encryption (HE) [15,16,17,18] was proposed. Xie and Wang [4] applied the RSA (for Rivest, Shamir and Adleman) algorithm to achieve computational functions on encrypted location data. In addition, Paillier’s cryptosystem [19,20,21,22,23] is widely used as HE, due to its high efficiency and simplicity. It involves only one multiplication for each homomorphic addition and one exponentiation for each homomorphic multiplication. Li and Jung [5] combined the ciphertext-policy attribute-based encryption (CP-ABE) with Paillier’s cryptosystem to exert fine-grained access control over LBS. One cannot gain any information from the query if his/her identity attributes do not satisfy the access policy defined by the data publisher. In addition, the location distance computed over encrypted location is supported. However, the publisher must stay online to interact with queriers once requested. This is not practical, considering the limited power of smart phone.

To overcome the above problems, we propose a privacy-preserving LBS scheme for mobile sensing data. Here, a new encryption method on the basis of the RSA algorithm (The RSA algorithm is a commonly adopted public key cryptography algorithm. It is named after the three mathematicians who developed it: Rivest, Shamir and Adleman. The security of this encryption algorithm is based on the hardness of the factoring problem. We will present this algorithm in Section 3.2.) and CP-ABE scheme is designed. Our proposed scheme has two advantages as follows:

- Secure sharing over location information with certain queriers. Our scheme achieves that location information is visible to specific queriers, while kept secret from others.
- Efficient and privacy-preserving location distance compute and compare. The location distance compute and compare are two of the most common functions in LBQ, i.e., what is the distance between the publisher’s and querier’s locations, or whether the distance is less than 100 m. Compared with the privacy-preserving location query protocol (PLQP) scheme [5], we make better use of the powerful energy in the mobile cloud, by the mobile cloud undertaking most of the computing overhead, such that the computation cost at the querier is very low.

The main contributions of our paper are outlined as follows:

1. A novel mobile sensing service system is constructed for privacy-preserving LBS.
2. This paper designs a novel encryption method on the basis of the RSA algorithm and CP-ABE scheme, so that the mobile cloud can process LBS over encrypted location information and only authorized queriers can get the query results.

The rest of this paper is organized as follows. Section 2 discusses the related work. Section 3 presents some preliminaries. Section 4 describes our system models. Section 5 gives the detailed design of our privacy-preserving LBS scheme for mobile sensing data. Section 6 analyzes the security of our proposed scheme. Section 7 shows the performance evaluation by experiments. Finally, Section 8 concludes this paper.

## 2. Related Work

Our paper designs a privacy-preserving LBS scheme for mobile sensing data. The related work mainly includes two aspects, i.e., privacy-preserving LBS and the access control technique.

### 2.1. Privacy-Preserving LBS

The k-anonymity technique has been widely used to achieve user location privacy in LBS. The basic idea is to remove some features, such that each item is not distinguishable among other k−1 items. It can ensure that a user can be identified with a probability of at most 1/k.

Kido et al. [24] proposed an anonymous communication technique for LBS to protect location privacy using dummies. Duckham and Kulik [25] presented a privacy-preserving location query algorithm by using the obfuscation method and vague location information of the user. Chow et al. [26] proposed a distributed k-anonymity model and a peer-to-peer spatial cloaking algorithm for the anonymous location-based services. Mokbel [27] proposed a location-obfuscation method that allows the server to record the real identifier of the user, but decreases the precision of the location information to protect the location privacy. Bamba et al. [28] proposed fast and effective location cloaking algorithms for location k-anonymity and location l-diversity in a mobile environment. Gedik and Liu [29] applied the personalized k-anonymity model to protect the location privacy of the user. Shankar et al. [30] proposed a fully-decentralized and autonomous k-anonymity-based system for location-based queries. Xue et al. [31] introduced the concept of location diversity, which improves spatial k-anonymity by ensuring that each query can be associated with at least *l* different semantic locations.

All of the above solutions can be applied to LBS. However, their techniques do not allow the cloud to search encrypted data. Therefore, they cannot be used for outsourced LBS where LBS data in the cloud are encrypted.

Li and Jung [5] designed a suite of fine-grained privacy-preserving location query protocols (PLQP) by applying Paillier’s cryptosystem [32,33]. It can solve the privacy issues in existing LBS applications. However, once there is an LBS request, the PLQP needs very frequent interaction between the publisher and the querier and much computation cost. In mobile sensing service systems, most queriers access the social networks via smart phones. The smart phones have weak power. Hence, it is unacceptable for the publishers to stay online always.

Shao, Lu and Lin [8] proposed a FINEframework based on the CP-ABE scheme. In this framework, LBS data are outsourced to a cloud server after encryption. Although the framework can ensure the confidentiality of LBS data, their search patterns will lead to the leakage of user location privacy, because their trapdoors generated from the locations are steady, which means trapdoors are always the same for the same location. It is easy for an attacker to count the frequency of a specific trapdoor and identify the known locations. In addition, this method is not efficient due to the low efficiency of the public encryption.

### 2.2. Access Control

Recently, the ABE scheme has been widely used to exert access control for LBS in the mobile cloud. Li and Jung [5] introduced CP-ABE to exert fine-grained access control over the location queries. One cannot gain any information from the query if his/her identity attributes do not satisfy the access policy defined by the data publisher. Shao, Lu and Lin [8] also employed CP-ABE in designing their FINE framework to achieve fine-grained access control over location-based service data.

The ABE scheme enables fine-grained access control over encrypted data using access policies and associates attributes with private keys and ciphertexts [34,35,36,37]. It was first proposed by Sahai and Waters [38], later extended to the key-policy ABE (KP-ABE) by Goyal et al. [39] and the CP-ABE by Bethencourt et al. [40]. In KP-ABE, the ciphertext is associated with an attribute set, and the user secret key is associated with an access policy over attributes. The user can decrypt the ciphertext if and only if the attribute set of the ciphertext satisfies the access policy specified in his/her secret key. The encryptor exerts no control over who has access to the data that he/she encrypts. In CP-ABE, the ciphertext is associated with an access policy over attributes, and the user secret key is associated with an attribute set. The user can decrypt the ciphertext if and only if the attribute set of his/her secret key satisfies the access policy specified in the ciphertext. The encryptor is able to decide who should or should not have access to the data that he/she encrypts. In our system model, CP-ABE is more suitable than KP-ABE because it enables the data publishers to determine an access policy over the outsourced location data, as studied by Li and Jung in [5].

## 3. Preliminaries

This section briefly describes some preliminaries used in our work, including the bilinear map, the RSA algorithm, CP-ABE and the access tree.

### 3.1. Bilinear Map

Let $G_{0}$ be a multiplicative cyclic group of prime order *p* and $g_{0}$ be its generator. The bilinear map *e* is defined as: $e:G_{0}\times G_{0}\to G_{T}$, where $G_{T}$ is the codomain of *e*. The bilinear map *e* has the following properties:

- Bilinearity: $\forall u,v\in G_{0}$ and $a,b\in Z_{p}$, $e\left(u^{a},v^{b}\right)=e{\left(u,v\right)}^{ab}$
- Symmetry: $\forall u,v\in G_{0}$, $e\left(u,v\right)=e\left(v,u\right)$.
- Non-degeneracy: $e\left(g_{0},g_{0}\right)\neq 1$.

**Definition** **1** (discrete logarithm assumption). *The discrete logarithm assumption in group $G_{0}$ of prime order p with generator $g_{0}$ is defined as follows: for any probabilistic polynomial-time (PPT) algorithm A, the probability that $\mathrm{Pr}\left[A\left(g_{0},g_{0}^{a}\right)=a\right]$ is negligible, where $g_{0},g_{0}^{a}\in G_{0}$, and $a\in Z_{p}$.*

**Definition** **2** (decisional Diffie–Hellman (DDH) problem). *The decisional Diffie–Hellman (DDH) problem in group $G_{0}$ of prime order p with generator $g_{0}$ is defined as follows: on input $g_{0},g_{0}^{a},g_{0}^{b},g_{0}^{c}=g_{0}^{ab}\in G_{0}$, where $a,b,c\in Z_{p}$, decide whether c=ab or c is a random element.*

**Definition** **3** (decisional bilinear Diffie–Hellman (DBDH) problem). *The decisional bilinear Diffie–Hellman (DBDH) problem in group $G_{0}$ of prime order p with generator $g_{0}$ is defined as follows: on input $g_{0},g_{0}^{a},g_{0}^{b},g_{0}^{c}=g_{0}^{ab}\in G_{0}$ and $e{\left(g_{0},g_{0}\right)}^{z}=e{\left(g_{0},g_{0}\right)}^{abc}\in G_{T}$, where $a,b,c\in Z_{p}$, decide whether z=abc or z is a random element.*

The security of many ABE schemes relies on the discrete logarithm assumption. The research also assumes that no PPT algorithm can solve the DDH and DBDH problems with non-negligible advantage. This assumption is reasonable since in a large number field, it is widely recognized that discrete logarithm problems (DLP) are as hard as described in Definition 1. Therefore, *a* is not deducible from $g_{0}^{a}$, even if $g_{0}$ is publicly known.

### 3.2. RSA: Public Key Cryptography Algorithm

RSA is a commonly-adopted public key cryptography algorithm [41]. It is the first and still most widely-used asymmetric algorithm. RSA is named after the three mathematicians who developed it, Rivest, Shamir and Adleman. The public/private key pair of RSA is computed in Algorithm 1, where $GenModulus\left(1^{N}\right)$ is a function used to output a composite modulus *n* along with its two *N*-bit prime factors; ϕ is Euler’s totient function; gcd is a function used to compute the greatest common divisor for two numbers.

The RSA encryption scheme includes three algorithms as follows:

1. $KeyGen\left(1^{N}\right)\to pk,sk$: takes security parameter $1^{N}$ as input and outputs a public/private key pair, denoted as $pk=\left(n,e\right)$ and $sk=\left(p,q,d\right)$, respectively, by executing Algorithm 1.
2. $Enc\left(m,pk\right)\to c$: on input, a public key $pk=\left(n,e\right)$ and a message $m\in Z_{n}^{*}$ compute the ciphertext as $c=m^{e}\;\mathrm{mod}\;n$.
3. $Dec\left(c,sk\right)\to m$: on input, a private key $sk=\left(p,q,d\right)$ and a ciphertext $c\in Z_{n}^{*}$ compute the message as $m=c^{d}\;\mathrm{mod}\;n$.

The security of the RSA encryption scheme relies on the hardness of the factoring problem. If an adversary can factorize *n*, then he/she can compute $\phi \left(n\right)=\left(p-1\right)\left(q-1\right)$ and obtain the secret key *d* by utilizing the Euclidean algorithm. However, factoring a large number is still a hard problem. The proper choice of the modulus n=pq can guarantee the security of RSA encryption scheme.

**Algorithm 1** KeyGen.**Input:** $1^{N}$: security parameter;**Output:** pk,sk: a public/private key pair;1: $\left(n,p,q\right)\leftarrow GenModulus\left(1^{N}\right)$;2: $\phi \left(n\right)=\left(p-1\right)\left(q-1\right)$;3: Find *e*, such that $gcd\left(e,\phi \left(n\right)\right)=1$, where $1<e<\phi \left(n\right)$;4: Compute $d=e^{-1}\;\mathrm{mod}\;\phi \left(n\right)$ **return**
$pk=\left(n,e\right)$, $sk=\left(p,q,d\right)$.

### 3.3. CP-ABE

In the CP-ABE, the private key is distributed to users by the trusted authority (TA) only once. The keys are identified with a set of descriptive attributes, and the encryptor specifies an encryption policy using an access tree, so that those with private keys the satisfy it can decrypt the ciphertext.

### 3.4. Access Tree $T_{P}$

**Figure 2:**
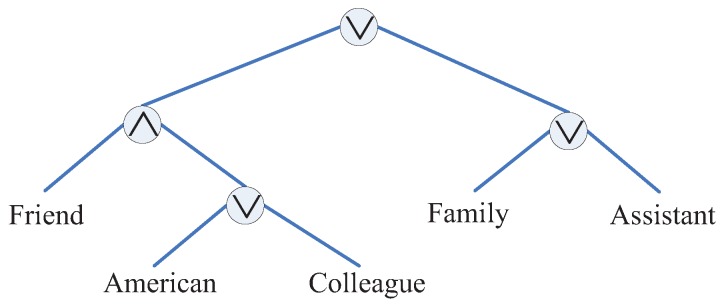
An example of the access tree.

In CP-ABE, the encryption policy is described with a tree called access tree $T_{p}$. Each non-leaf node of the tree is a threshold gate, and each leaf node is described by an attribute. An example is shown in Figure 2.

In this paper, a publisher’s location information is set visible to certain kinds of users. For example, in Figure 1, Alice’s location information is only accessible to her friends. If a user’s attributes satisfy $T_{P}$, he/she is granted with the access privilege. Simultaneously, he/she also can obtain the results of LBS provided by the mobile cloud. By doing so, we can control the visibility of the publisher’s location information.

Given a node *u* in the $T_{P}$, |Children(u)| is the number of the node *u*’s children nodes, and $k_{u}$ is its threshold value $0<k_{u}\leq \left|Children\left(u\right)\right|$. The node *u* is assigned a true value if at least $k_{u}$ child nodes have been assigned a true value. Specially, the node becomes an ORgate when $k_{u}=1$ or an ANDgate when $k_{u}=\left|Children\left(u\right)\right|$.

The access tree is described by a set of polynomials, as shown in Algorithm 2. In the access tree $T_{P}$, the node value of the gate is recovered if and only if the values of at least $k_{u}$ child nodes are recovered, which is performed in a recursive manner. The notations for the access tree is explained in Table 1.

**Algorithm 2** Access Tree Description.**Input:**  $T_{P}$: an access tree;**Output:**  $\left\{s,q_{lf}\left(0\right)|lf\;\mathrm{is}\;a\;\mathrm{leaf}\;\mathrm{node}\;\mathrm{of}\;T_{P}\right\}$: $s\in Z_{p}$ is a randomly-picked secret integer; 1: **for all**
*u* in $T_{P}$
**do** 2:   define a polynomial $q_{u}\left(x\right)=\sum_{i=0}^{k_{u}-1}a_{ui}x^{i}$, where the coefficients $a_{ui}$ are undetermined; 3: **end for** 4: Pick a random integer *s* 5: Set $a_{R0}=q_{R}\left(0\right)=s$, where *R* is the root node of $T_{P}$; 6: Set other coefficients of $q_{R}\left(x\right)$, i.e., $a_{Ri},i=1,2,\ldots ,k_{R}-1$, by randomly-picked secret integers; 7: From top to bottom, set all of the coefficients of other nodes (except for the root node) that satisfy the following equation;
$$q_{u}\left(0\right)={q_{parent}}_{\left(u\right)}\left(index\left(u\right)\right).$$  **return**
$\left\{s,q_{lf}\left(0\right)|lf\;\mathrm{is}\;a\;\mathrm{leaf}\;\mathrm{node}\;\mathrm{of}\;T_{p}\right\}$.

## 4. System Model, Threat Model, Location Assumption and Problem Formulation

### 4.1. System Model

**Figure 3:**
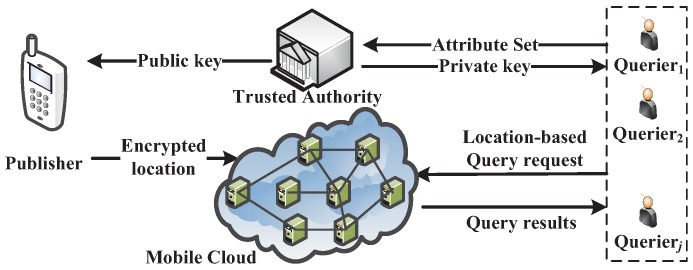
The mobile sensing service system.

Our mobile sensing service system mainly consists of four entities, as shown in Figure 3: mobile cloud, a publisher, many queriers and TA.

The mobile cloud provides LBS via mobile applications or social network applications based on the collected location data. Its main work is to store and process ciphertext.

A publisher contributes his/her location data to the mobile cloud, via smart phone, iPad, etc. Before uploading the data, the publisher first obtains the public key from the TA and determines the access tree. Then, he/she uses the public key and access tree to encrypt his/her location information. Afterwards, the encrypted location information is uploaded to the mobile cloud for storage and sharing. In addition, we assume the origin of a packet is successfully hidden, which is out of this paper’s scope, and can be trivially prevented by employing anonymized network protocols [42].

Many queriers submit LBS query requests to the mobile cloud over the collected cloud data. However, only authorized queriers can obtain plain query results.

TA is assumed to have powerful computation abilities. At the setup phase, the TA computes its own master key and the system-wide public parameter. The master key is used to generate the private key for the queriers, and the public key is used to process system-wide operations.

### 4.2. Threat Model

We assume that the mobile cloud is “honest but curious”. Specifically, it acts in an “honest” fashion and correctly follows the designated protocol specification. However, it is “curious” to infer and analyze the stored data and queriers’ query requests to harvest additional information to gain illegal profits.

The queriers are curious about the confidential information, which is outside of their privileges. They may also collude with the mobile cloud.

### 4.3. Location Assumption

As described in [5], the ground surface can be assumed to be a plane, and every user’s location is mapped to an Euclidean space with integer coordinates (with meters as the unit). The Euclidean distance between two locations $X=(x_{1},x_{2},x_{3})$ and $Y=(y_{1},y_{2},y_{3})$ is computed as:

$$dist\left(X,Y\right)=\left|X-Y\right|=\sqrt{\sum_{i=1}^{3}{\left(x_{i}-y_{i}\right)}^{2}}.$$

As for a real location on the surface of the Earth, we know that the relationship between the surface distance SD(X,Y) and the Euclidean distance dist(X,Y) is as follows:

$$SD\left(X,Y\right)=2\arcsin\left(\frac{dist\left(X,Y\right)}{2R}\right)\cdot R,$$

where the Earth is assume to be a sphere with radius *R* meters. Hence, it is easy to compute SD(X,Y) from dist(X,Y). To check if the surface distance satisfies certain conditions, we can convert it to check if the Euclidean distance is satisfying the corresponding conditions. For example, dist(X,Y)≤τ is equivalent as:

SD(X,Y)≤2Rarcsin(τ/2R).

For brevity, in this paper, we will focus on the Euclidean distance instead of the surface distance.

### 4.4. Problem Formulation

Assume that the querier Q’s location information is $X=\left(x_{1},x_{2},x_{3}\right)$, and the publisher P’s location information $Y=\left(y_{1},y_{2},y_{3}\right)$ is embedded in the published data. According to *Q*’s attributes set $S_{Q}$, the publisher P determines whether the querier Q can enjoy the LBS related to P’s location. Afterwards, the authorized querier will obtain the corresponding LBS results provided by the mobile cloud.

In this paper, we design a privacy-preserving LBS scheme for mobile sensing data, where the location publisher can determine who can decrypt the ciphered LBS results provided by the mobile cloud. Moreover, no confidential location information is leaked to the mobile cloud and unauthorized users during the LBS processing. Here, the LBS mainly includes two basic types: location distance compute and compare. The proposed scheme includes five main algorithms, as follows:

- $Setup(1^{N})\to MK,PK$
  This algorithm takes a security parameter $1^{N}$ as input. The TA executes this algorithm to compute its own master key MK and a system-wide public parameter PK.
- $Encrypt\left(PK,Y,T_{P},K_{Y}\right)\to Y_{e}$
  This algorithm takes as input the public parameter PK, the publisher’s location information $Y=\left(y_{1},y_{2},y_{3}\right)$, an access tree $T_{P}$ determined by the publisher and an encryption key $K_{Y}$. It will encrypt the location *Y*, so that a querier can enjoy the LBS over location *Y* if and only if his/her attributes satisfy the access tree $T_{P}$.
- $KeyGenerate\left(MK,PK,S_{Q}\right)\to {SK}_{Q}$
  This algorithm takes as input the TA’s master key MK, the public parameter PK and a querier’s attribute set $S_{Q}$. It enables the querier to interact with the TA and to obtain a secret key $SK_{Q}$.
- $Verify\left(PK,{SK}_{Q},S_{Q},Y_{e}\right)\to W^{s}$ or ⊥
  This algorithm enables an authorized querier to obtain a critical secret parameter $W^{s}$, which is the key to decrypt the ciphered query results provided by the mobile cloud.
- $Operate(Y_{e},W^{s},X)\to answer$
  In this protocol, firstly, a querier encrypts his/her location *X* as $X_{e}$ using $W^{s}$, then the mobile cloud operates over the encrypted locations $Y_{e}$ and $X_{e}$ to compute a ciphered query result. In the end, the querier uses $W^{s}$ to decrypt the ciphered result as answer.

## 5. Our Proposed Scheme

In this section, we will present the scheme design in detail.

A. $Setup(1^{N})\to MK,PK$

This algorithm takes a security parameter $1^{N}$ as the input, and gives the TA’s master key MK and a system-wide public parameter PK as the output, as shown in Algorithm 3.

By Algorithm 3, the TA chooses and publishes a bilinear group $G_{0}$ of prime order *p* with generator $g_{0}$, then randomly and secretly picks $v_{0}\in Z_{p}$. Finally, the TA computes the master key MK and the public key PK.

B. $Encrypt\left(PK,Y,T_{P},K_{Y}\right)\to Y_{e}$

Before uploading the location data, the publisher executes this algorithm to encrypt the sensitive location information $Y=\left(y_{1},y_{2},y_{3}\right)$. In addition, she/he determines the access tree $T_{P}$ to exert access control on the location information. The ciphertext $Y_{e}$ includes two parts: $Y_{e}^{I}$ and $Y_{e}^{II}$. The encryption procedure consists of three main steps.

1. Pick a symmetric encryption key $K_{Y}=\langle x,m\rangle$, where 0<x,m<n, *n* is generated by $GenModulus\left(1^{N}\right)$, as shown in Section 3.2.
2. Compute:
  (1) $$y_{ei}=y_{i}\cdot {\left(mg^{\left(x\;\mathrm{mod}\;\phi \left(n\right)\right)}\right)}^{-1}modn,i=1,2,3.$$
  Here, *g* is co-prime with *n*; $\phi \left(n\right)$ is Euler’s totient function of *n*. We will omit “$\;\mathrm{mod}\;\phi \left(n\right)$” in the following expressions with an assumption that the exponent of the above formula is computed in modular $\phi \left(n\right)$.
3. Execute Algorithm 2, and obtain $\left\{s,q_{lf}\left(0\right)|lf\;\mathrm{is}\;a\;\mathrm{leaf}\;\mathrm{node}\;\mathrm{of}\;T_{p}\right\}$. Then, $Y_{e}^{I}$ and $Y_{e}^{II}$ are computed by Equations (2) and (3). In $Y_{e}^{II}$, $C_{u}$ and ${C^{\prime }}_{u}$ represent the attribute values in the specified access tree.
  (2) $$Y_{e}^{I}=\left\{\langle y_{e1},y_{e2},y_{e3}\rangle ,\langle x+W^{s},m\cdot W^{s}\rangle \right\},$$
  (3) $$Y_{e}^{II}=\langle T_{p},{\left\{C_{u}=g_{0}^{q_{u}\left(0\right)},{C^{\prime }}_{u}=H{\left(att\left(u\right)\right)}^{q_{u}\left(0\right)}\right\}}_{u\in leaf(T_{P})},C=g_{0}^{s}\rangle .$$

Finally, the publisher stores $Y_{e}=\left\{Y_{e}^{I},Y_{e}^{II}\right\}$ in the mobile cloud for sharing them with some queriers. In this system, $Y_{e}$ can be downloaded by every querier. However, only authorized queriers can obtain the plain location information *Y*, and the query results of location distance compute and compare.

**Algorithm 3** Setup.**Input:**  $1^{N}$: security parameter;**Output:**  MK: TA’s master key;  PK: public parameter; 1: Choose a bilinear group $G_{0}$ of prime order *p* with generator $g_{0}$; 2: Pick $v_{0}\in Z_{p}$ randomly and secretly; 3: Compute the master key $MK=g_{0}^{v_{0}}$; 4: Compute $W=e{\left(g_{0},g_{0}\right)}^{v_{0}}$; 5: Set $PK=\left\{G_{0},g_{0},W\right\}$;  **return**
MK,PK.

C. $KeyGenerate\left(MK,PK,S_{Q}\right)\to {SK}_{Q}$

When a new querier Q, with attribute set $S_{Q}$, requests to join the system, TA executes this algorithm to generate *Q*’s secret key. This algorithm is composed of two steps, as follows:

1. Attribute key generate: TA randomly picks $d_{0}\in Z_{p}$ and computes:
  (4) $$D=MK\cdot g_{0}^{d_{0}}.$$
  For any attribute $i\in S_{Q}$, TA randomly picks $r_{i}\in Z_{p}$ and computes the partial private key as:
  (5) $$D_{i}=H{\left(i\right)}^{r_{i}}\cdot g_{0}^{d_{0}},$$
  (6) $${D^{\prime }}_{i}=g_{0}^{r_{i}}.$$
  where H(i) is the hash value of attribute *i*.
2. Key aggregate: The secret key is generated by aggregating *D*, $D_{i}$, and ${D^{\prime }}_{i}$ as:
  (7) $${SK}_{Q}=\left\{D,\forall i\in S_{Q}:D_{i},{D^{\prime }}_{i}\right\}.$$
  The above procedures are described in Algorithm 4.

**Algorithm 4** KeyGenerate.**Input:**  MK: TA’s master key;  PK: the public parameter;  $S_{Q}$: a querier’s attribute set;**Output:**  $SK_{Q}$: a secret key; 1: Compute $D=MK\cdot g_{0}^{d_{0}}$, where $d_{0}\in Z_{p}$; 2: **for all**
$i\in S_{Q}$
**do** 3:   Compute $D_{i}=H{\left(i\right)}^{r_{i}}\cdot g_{0}^{d_{0}}$, ${D^{\prime }}_{i}=g_{0}^{r_{i}}$, where $r_{i}\in Z_{p}$; 4: **end for**  **return**
${SK}_{Q}=\left\{D,\forall i\in S_{Q}:D_{i},{D^{\prime }}_{i}\right\}$.

D. $Verify\left(PK,{SK}_{Q},S_{Q},Y_{e}\right)\to W^{s}$ or ⊥

**Figure 4:**
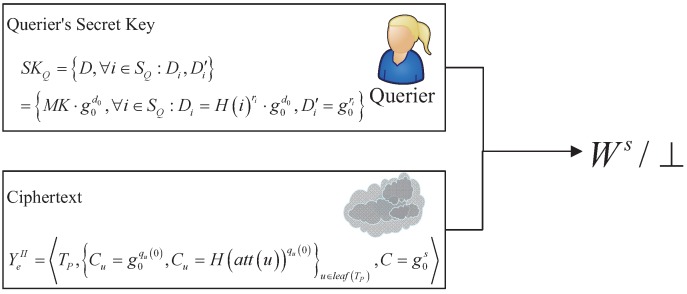
The overview of verification process.

By executing this algorithm, only authorized querier can obtain the secret parameter $W^{s}$, which will be used to decrypt the ciphered query results. Otherwise, it will output ⊥. Figure 4 shows the main overview of this algorithm.

Firstly, a recursive algorithm $DecryptNode\left(Y_{e}^{II},{SK}_{Q},S_{Q},u\right)$ is defined as Algorithm 5, where *u* stands for a node in the access tree $T_{P}$.

Then, the querier recursively calls $DecryptNode\left(Y_{e}^{II},{SK}_{Q},S_{Q},R\right)$ from the root node *R*, and obtains ${par}_{R}=e{\left(g_{0},g_{0}\right)}^{s\cdot d_{0}}$. At last, he/she can get the secret output $W^{s}$ by computing Equation (8).

(8) $$\frac{e\left(C,D\right)}{par_{R}}=W^{s}$$

E. $Operate(Y_{e},W^{s},X)\to answer$

In this protocol, firstly, the authorized querier uses $W^{s}$ to encrypt his/her location *X* as $X_{e}$, then the mobile cloud operates over $Y_{e}$ and $X_{e}$ to compute a ciphered location distance or to test whether the distance between these two locations is far or not. If necessary, the querier will use $W^{s}$ to decrypt the ciphered result as answer.

In our scheme, we consider these two types of operations: location distance compute and location distance compare, i.e., what the distance between these two locations is or whether the distance is far or not. They are two basic LBS.

**Algorithm 5** DecryptNode.**Input:**  $Y_{e}^{II}$: defined in Equation (3)  ${SK}_{Q}$: the querier Q’s secret key;  $S_{Q}$: the querier Q’s attribute set;  *u*: a node in the access tree $T_{P}$;**Output:**  $par_{u}$: a secret parameter;    or ⊥ 1: **if**
*u* is a leaf node **then** 2:   Set i=att(u); 3:   **if**
$i\in S_{Q}$
**then** 4:     Compute
$$par_{u}=\frac{e\left(D_{i},C_{u}\right)}{e\left({D^{\prime }}_{i},{C^{\prime }}_{u}\right)}=e{\left(g,g\right)}^{q_{u}\left(0\right)\sum d_{k}};$$ 5:   **else**
**return**
$par_{u}=⊥$; 6:   **end if** 7: **else** 8:   Define $F_{u}=null$; 9:   **for all**
z∈Children(u)
**do** 10:     Compute $par_{z}=DecryptNode\left(Y_{e}^{II},{SK}_{Q},S_{Q},z\right)$; 11:     **if**
$par_{z}\neq ⊥$
**then** 12:       Update $F_{u}=F_{u}\cup \left\{par_{z}\right\}$; 13:     **end if** 14:   **end for** 15:   **if**
$|F_{u}|<k_{u}$
**then**
**return**
$par_{u}=⊥$; 16:   **else** 17:     Compute $par_{u}=e{\left(g_{0},g_{0}\right)}^{q_{u}\left(0\right)d_{0}}$ using $F_{u}$ by polynomial interpolation method; 18:   **end if** 19: **end if**  **return**
$par_{u}$.

Here, the locations of the publisher and the querier are $X=\left(x_{1},x_{2},x_{3}\right)$ and $Y=\left(y_{1},y_{2},y_{3}\right)$, respectively. Additionally, the publisher’s location data $Y=\left(y_{1},y_{2},y_{3}\right)$ have been encrypted as $Y_{e}=\left\{Y_{e}^{I},Y_{e}^{II}\right\}$ by Equations (2) and (3) and stored in the mobile cloud. The querier encrypts his/her location *X* using Equation (9).

(9) $$\begin{array}{cc} & X_{e}=\left(x_{e1},x_{e2},x_{e3}\right)\\ & =\left(x_{1},x_{2},x_{3}\right)\cdot W^{s}\cdot g^{W^{s}}\\ & =\left(x_{1}\cdot W^{s}\cdot g^{W^{s}},x_{2}\cdot W^{s}\cdot g^{W^{s}},x_{3}\cdot W^{s}\cdot g^{W^{s}}\right) \end{array}$$

Next, we will present how to perform these two operations.

1. Location distance compute: 

We know that the distance between the publisher and the querier can be computed as:

(10) $$dis=\sqrt{\sum_{i=1}^{3}{\left(x_{i}-y_{i}\right)}^{2}}.$$

The querier encrypts his/her location $X=\left(x_{1},x_{2},x_{3}\right)$ as $X_{e}$ by Equation (9) and sends $X_{e}$ to the mobile cloud. Then, the mobile cloud executes Algorithm 6 to compute the ciphered location distance between $X_{e}$ and $Y_{e}$. For simplicity and convenience of presentation, we will denote $W^{s}\cdot g^{W^{s}}$ as Δ in the following.

**Algorithm 6** DistanceCompute.**Input:**  $X_{e}$: the ciphertext of a querier’s location *X*;  $Y_{e}$: the ciphertext of a publisher’s location *Y*;**Output:**  ${dis}_{e}$: the ciphertext of the distance between *X* and *Y*; 1: Obtain $m^{\prime }=m\cdot W^{s}$ and $x^{\prime }=x+W^{s}$ from the $Y_{e}^{I}$ ; 2: Compute ${K^{\prime }}_{Y}=m^{\prime }\cdot g^{x^{\prime }}\;\mathrm{mod}\;n$; 3: Compute ${dis}_{e}=\sqrt{\sum_{i=1}^{3}{\left(x_{ei}-y_{ei}\cdot {K^{\prime }}_{Y}\right)}^{2}}$;    **return**
${dis}_{e}$;

From Algorithm 6 and Equation (1), we know that for i=1,2,3,

$$\begin{array}{cc} & x_{ei}-y_{ei}\cdot {K^{\prime }}_{Y}\\ & =x_{i}\cdot \Delta -y_{i}\cdot {\left(mg^{x}\right)}^{-1}\cdot {K^{\prime }}_{Y}\\ & =(x_{i}-y_{i})\cdot \Delta \end{array}$$

Thus,

(11) $$\begin{array}{cc} & {dis}_{e}=\sqrt{\sum_{i=1}^{3}{\left(x_{ei}-y_{ei}\cdot {K^{\prime }}_{Y}\right)}^{2}}=\sqrt{\sum_{i=1}^{3}{\left(x_{i}-y_{i}\right)}^{2}}\cdot \Delta =dis\cdot \Delta \end{array}$$

After executing Algorithm 6, the mobile cloud sends $dis_{e}$ to the querier. Finally, the querier can get the plain distance dis by computing Equation (12).

(12) $$dis=dis_{e}\cdot \Delta^{-1}.$$

During the execution, all that the mobile cloud processes is the ciphertext.

2. Location distance compare: 

The querier wants to know whether the location distance is within a threshold value *τ*. He/she encrypts the *τ* as $\tau_{e}$, using Equation (13):

(13) $$\tau_{e}=\tau \cdot \Delta .$$

Then, the querier sends $X_{e}$ and $\tau_{e}$ to the mobile cloud. The mobile cloud executes Algorithm 7 to compute whether the distance between *X* and *Y* is less than *τ*.

From Equation (11), we know that ${dis}_{e}=dis\cdot \Delta$. Since Δ is always positive, it will not change the compare result between dis and *τ*. Thus, the mobile cloud can give out the comparison results through Algorithm 7 directly.

**Algorithm 7** DistanceCompare.**Input:**  $X_{e}$: the ciphertext of a querier’s location *X*;  $Y_{e}$: the ciphertext of a publisher’s location *Y*;  $\tau_{e}$: the ciphered compare parameter *τ*;**Output:**  true or false; 1: Execute $dis_{e}=DistanceCompute\left(X_{e},Y_{e}\right)$; 2: **if**
$dis_{e}-\tau_{e}\leq 0$
**then return**
true; 3: **end if**  **return**
false;

## 6. Security Analysis

In our system, the publisher can authorize the queriers that he/she knows or not, such as his/her friends or someone who has similar interests. Hence, the queries may include attackers. If so, it is easy for the attacker to get a certain plaintext/ciphertext pair. Thus, our scheme has to be secure against the chosen plaintext attack. Next, we will prove it.

**Theorem** **1.** Our scheme is secure against CPA.

**Proof of Theorem** **1.** Assume an attacker obtains $Y=\{y_{1},y_{2},y_{3}\}$ and its ciphertext $Y_{e}$. From Equation (1), we get that:

(14) $$mg^{x}=\frac{y_{i}}{y_{ei}},i=1,2,3.$$

Assume that $B=mg^{x}$. It is easy to compute *B*. However, it is difficult to get the proper 〈x,m〉 from *B*. If *m* is the power of *g*, it comes down obviously to the discrete logarithm problem. If *m* is not the power of *g*, i.e., $m=m^{\prime }g^{x^{\prime }}$, where $m^{\prime }$ is co-prime with *g* and $x^{\prime }\geq 0$, then $B=m^{\prime }g^{x^{\prime }+x}$. Even though the attacker can solve this equation, there are multiple solutions $x^{\prime },x$ for $x^{\prime }+x$. Let alone, it is even hard to solve $m,x^{\prime }+x$ from *B*, which is as intractable as the discrete logarithm problem. As a consequence, the attacker cannot deduce the encryption key, as well as the secret parameter $W^{s}$ from $Y_{e}^{I}$. As described in Section 5, we know that secret parameter $W^{s}$ is the key to decrypt the query result. Thus, the attacker cannot decrypt extra confidential information apart from the already known $Y=\{y_{1},y_{2},y_{3}\}$. In conclusion, our scheme is secure against CPA.  ☐

## 7. Experiment

As described in Section 2.1, the PLQP [5] is a suit of protocols supporting privacy-preserving LBS in mobile application. It has high efficiency and achieves fine-grained control by exerting the CP-ABE scheme, which is similar to our work. Thus, in this section, we will compare our scheme with the PLQP scheme [5] for evaluating the performance of our proposed scheme. The algorithms are implemented using the BigInteger library on a Windows 8.1 system with Intel CORE i7-4500U CPU@2.40 GHz and 8.00 G RAM. We have 10 tests in this experiment. Additionally, in each test, we use 1000 pairs of random locations for the publisher and the querier, respectively. We present the average results for each test in the following figures.

### 7.1. The Time Cost in Our Scheme

**Figure 5:**
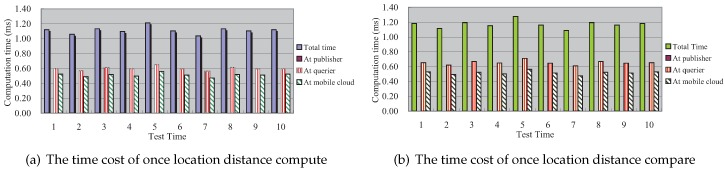
The time cost in our scheme.

Figure 5 shows the detailed time cost for once location distance compute and location distance compare, respectively. It is obvious that the time cost at the publisher is always zero, which meets the Operatealgorithm in Section 5. The query processing work can be done with no need for the publisher’s help.

### 7.2. The Comparison of The Time Cost between Our Scheme and the PLQP Scheme

**Figure 6:**
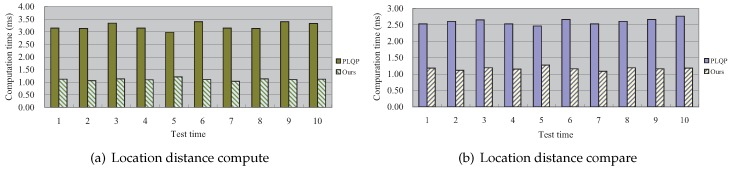
The comparison of the total time cost between our scheme and PLQP.

**Figure 7:**
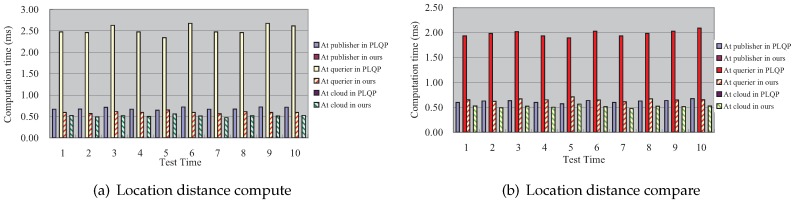
The comparison of each entity’s time cost between our scheme and PLQP.

**Figure 8:**
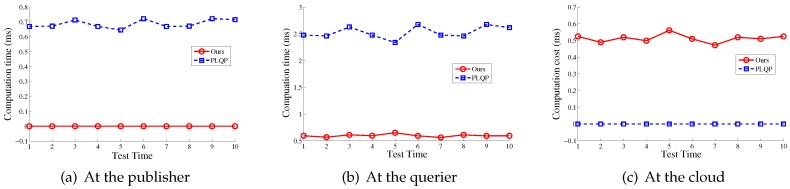
The time comparison of performing location distance compute between our scheme and PLQP.

**Figure 9:**
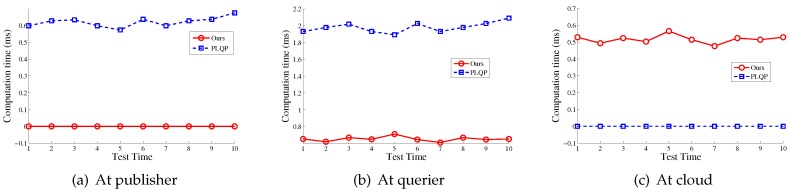
The time comparison of performing location distance compare between our scheme and PLQP.

In Figure 6, we show the comparison results of the total time cost for the aforementioned two operations, respectively. It can be seen that our scheme is much more efficient than PLQP. Figure 7 shows the comparison results of the detailed time cost for the aforementioned two operations at the publisher, querier and mobile cloud, respectively. To be more clear, we also present the comparison results in Figure 8 and Figure 9. From these figures, we get three points:

- At the querier, the time cost in our scheme is much less that that in PLQP.
- At the publisher, the time cost in our scheme is zero.
- At the mobile cloud, the time cost in PLQP is zero.

In sum, our scheme takes better advantage of the mobile cloud than PLQP and outperforms the PLQP scheme in terms of query efficiency. Numerical information about the computation cost is also shown in Table 2.

## 8. Conclusions

In this paper, we propose a privacy-preserving LBS scheme for mobile sensing data, by exerting the RSA algorithm and CP-ABE scheme. Our proposed scheme can support the mobile cloud to perform efficient and privacy-preserving queries of location distance compute and compare on encrypted locations. Moreover, due to the application of CP-ABE, our scheme achieves fine-grained control over the sensitive location data, where only authorized queriers, whose attributes satisfy the corresponding access tree, can decrypt the ciphered query results provided by the mobile cloud. As a consequence, both the publisher’s and querier’s location information are kept secret from the mobile cloud and unauthorized users. Finally, the security analysis proves that it is secure against CPA, and the performance evaluation demonstrates its efficiency with experiments compared with the efficient PLQP.

## Figures and Tables

**Table 1:** The notations for access tree $T_{P}$.

Notation	Description
*u*	a node in $T_{P}$
$leaf(T_{p})$	the set of leaf nodes in $T_{P}$
Children(u)	the set of all the child nodes of *u*
|Children(u)|	the number of the node *u*’s child nodes
$k_{u}$	the threshold value of node *u*
$q_{u}\left(x\right)=\sum_{i=0}^{k_{u}-1}a_{ui}x^{i}$	the polynomial equation of node *u*, where $a_{ui}\in Z_{p},i=1,2,...,k_{u}-1$ are the coefficients
parant(u)	*u*’s parent node
index(u)	the index of node *u*

**Table 2:** The computation cost comparison.

Queries	Average Computation Time at the Querier (ms)		Average Computation Time at the Publisher (ms)		Average Computation Time at the Mobile Cloud (ms)	
	Ours	PLQP	Ours	PLQP	Ours	PLQP
Distance Compute	0.59850795	2.52919789	0	0.68694300	0.51267655	0
Distance Compare	0.65277693	1.98040717	0	0.62046427	0.51697024	0

## Abbreviations

The following abbreviations are used in this manuscript:

GPS	Global Positioning System
LBS	Location-based service
HE	Homomorphic encryption
ABE	Attribute-based encryption
CP-ABE	Ciphertext-policy attribute-based encryption
KP-ABE	Key-policy attribute-based encryption
AES	Advanced encryption standard
DES	Data encryption standard
PLQP	Privacy-preserving location query protocol
DLP	Discrete logarithm problem
PPT	Probabilistic polynomial-time
DDH	Decisional Diffie–Hellman
DBDH	Decisional Bilinear Diffie–Hellman
CPA	Chosen plaintext attack
TA	Trusted authority
